# Effect of microplastics on nasal and gut microbiota of high-exposure population: Protocol for an observational cross-sectional study

**DOI:** 10.1097/MD.0000000000030215

**Published:** 2022-08-26

**Authors:** Xiyu Zhang, Yuchi He, Ziyan Xie, Sihan Peng, Chunguang Xie, Heting Wang, Lu Liu, Jian Kang, Haipo Yuan, Ya Liu

**Affiliations:** a Hospital of Chengdu University of Traditional Chinese Medicine, TCM Regulating Metabolic Diseases Key Laboratory of Sichuan Province, China; b School of Clinical Medicine, Chengdu University of Traditional Chinese Medicine, China; c Department of Traditional Chinese Medicine, Sichuan Provincial People’s Hospital, University of Electronic Science and Technology of China, China.

**Keywords:** 16S rDNA sequencing, 8700 LDIR laser infrared imaging, environment, gut microbiota, microplastics, nasal microbiota

## Abstract

Microplastics have the characteristics of small size, high specific area, strong ability to adsorb pollutants, and difficult to degrade. They have become a major global environmental problem that humans urgently need to address. A balanced microecosystem is essential to human health. Animal studies have shown that long-term exposure to microplastics can change the characteristics of the microbiota in organisms, leading to respiratory, digestive, immune, and other system diseases. However, the current research on microplastics is still dominated by animal experiments, and the impact of microplastics on human health is still in its infancy, so relevant research is urgently needed. Twenty participants with high exposure to microplastics will come from a plastic factory in Chengdu, China. We will perform 16S rDNA sequencing on participants’ nasal secretions, and stool samples. Additionally, we will perform 8700 LDIR laser infrared imaging of environmental soil and air filter membrane samples. For comparison, we will also collect samples from 20 volunteers from an area with good environmental quality in Chengdu. To find out the potential predictors and to access the difference between the groups, statistical analysis will be performed in the end. The study will be the first observational cross-sectional study focusing on the effects of microplastics on nasal and gut microbiota of high-exposure population. The study is expected to provide reliable evidence to fill the gaps in the impact of microplastics on human health.

## 1. Introduction

The term “microplastic” was first used by Thompson in the literature in 2004 to describe tiny plastic particles existing marine environment.^[1]^ Microplastics is usually defined as plastic debris with size below 5 mm which comes from breaking of larger plastic objects (secondary microplastics) and tiny plastic particles (primary plastics) directly released by human activities.^[2]^ In the year of 2018, the total plastics released by the whole world was up to 359 million tons.^[3]^ And it’s not the first problem caused by the production, apply, and consumption of plastics. In fact, it has caused thousands of major environment problems since 1950s. As a result of wild use of plastics, microplastics now is ubiquitous. The presences of microplastics have been reported in ocean, freshwater, food, outdoor and indoor air, soil, and even the South Pole.^[4–8]^

Researchers found out that the nasal cavity is the key part of the inhalation of microplastics in the air, and the intestine is the main enrichment part of the microplastics after ingestion.^[3]^ As is known to all, a balanced microecosystem of the nasal cavity and intestines is essential to biological health. Microplastics can enter human body by ingestion and inhalation and cause diseases. Studies indicated that worker who is in high exposure to microplastics such as polypropylene (PP) and Polyamide at work shows increased risk of lung diseases.^[9]^ What’s more, the risk of PP flocked workers having respiratory symptoms increased 3.6 times compared with the control group, and subtle or minor interstitial lung diseases were also found within those workers.^[10]^ According to some animal studies, long-term exposure to microplastics would alter the characteristics of microbiota inside, which may lead to diseases in respiratory, digestive, and immune system.^[11–13]^

Animal studies have shown that long-term exposure to microplastics can change the characteristics of the microbiota in organisms, leading to respiratory, digestive, immune, and other system diseases. However, the current research on microplastics is still dominated by animal experiments, and the impact of microplastics on human health is still in its infancy, so relevant research is urgently needed. This study will conduct an observational cross-sectional study to investigate how microplastics affect the nasal and gut microbiota of high-exposure populations. We hope to build on these findings to provide solid evidence for filling the gaps in the effects of microplastics on human health.

## 2. Methods and analysis

### 2.1. Study design

**Figure 1.**
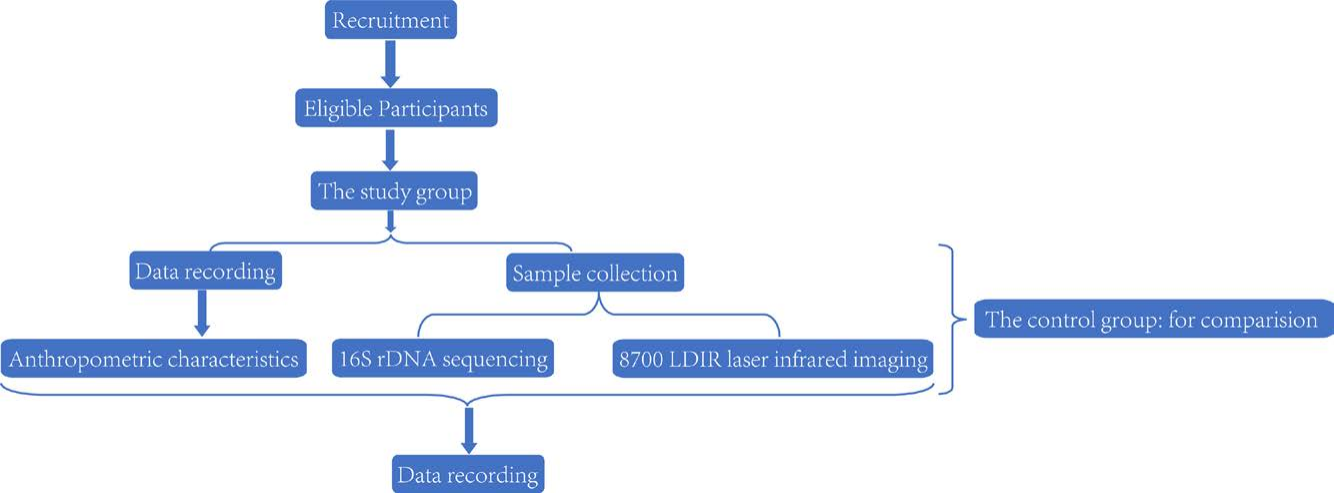
Study process: flowchart of study procedure.

**Figure 2.**
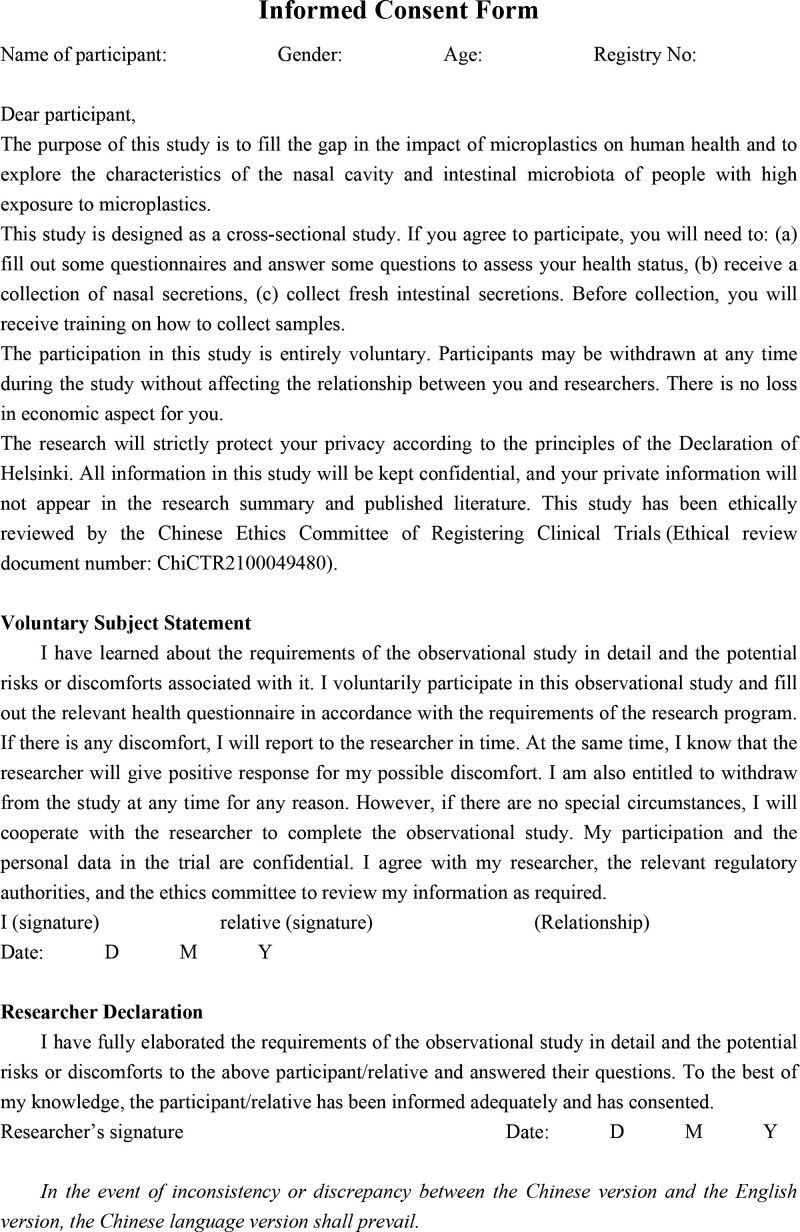
Informed consent form.

This study is designed as an observational cross-sectional study and was developed according to the Strengthening the Reporting of Observational Studies in Epidemiology (the STROBE checklist, Supplemental Digital Content, http://links.lww.com/MD/H79). A flowchart of this trial procedure is shown in Figure 1. The nasal secretions and feces of subjects exposed to microplastics will be collected and analyzed to determine the characteristics of the nasal and gut flora under high exposure of microplastics. Participants will sign a written informed consent form (Fig. 2; Informed Consent Form, Supplemental Digital Content, http://links.lww.com/MD/H80) and have a clear understanding of the purpose, study procedures, and all potential risks related to the study.

### 2.2. Study subject

The study is conducted at a plastic factory in Chengdu, China, which mainly produces plastic products including polyethylene, polyvinyl chloride, polytetrafluoroethylene, PP, and polystyrene. We will post recruitment information on intranet and bulletin boards of the factory. Volunteers should meet the inclusion and exclusion criteria below to officially become a participant of the study (inclusion and exclusion criteria for study enrollment detailed in Table 1).

**Table 1:** Inclusion and exclusion criteria for the study enrollment.

Inclusion criteria	Exclusion criteria
• Participants who voluntarily participate in this study and sign an informed consent form.	• History of systemic or nasal use of antibotics, antifungal, hormones, and other medications affecting the microecology of the flora within 3 months.
• Age: 18 to 65 yr, gender is not limited.	• History of systemic or nasal use of probiotics/probiotic products (including medications, yogurt, beverages) within 3 months.
• No nasal diseases such as nasal tumor, congenital malformation, and structural abnormality.	• Obviously mental disorders.
• No organic digestive system diseases such as peptic ulcer, inflammatory bowel diseases, gastrointestinal tumor.	• Long history of smoking.
• No serious primary diseases in cardiovascular, digestive, urinary, and hematopoietic system.	• Pregnancy and lactation.
• Factory workers with fixed working time ≥ 8 hr/d and continuous working years ≥ 3 yr.	

Members of the research group (Z.X.Y., P.S.H., W.H.T., L.Y.) will be involved in recruiting participants, all of whom are medical staff with physician certificates and will receive the necessary training to communicate with people and collect samples. The recruitment period is from July 2021 to July 2022, and each group is expected to recruit 60 participants.^[14]^ All information and data of participants will be confidential. Only members of the research group and principal investigator can have access to them. After the late data entry is completed, participants can log on to the website (http://www.medresman.org.cn/login.aspx) to query the details.

To clarify the characteristics of the microbiota of the study group, we will also collect samples and questionnaires from 20 volunteers from areas with good environmental quality in Chengdu. These volunteers will also meet the inclusion and exclusion criteria except that they have been living or working continuously for ≥6 months and the daily time is ≥8 hours per day within 1 kilometer from the center of the Huanhuaxi Park. Huanhuaxi Park is the largest open city forest and wetland park (32.32 ha) in Chengdu, China, with significantly better air quality index than other regions of Chengdu.

### 2.3. Sample size

Sample size was calculated by GPower 3.1 software, using *t* test: correlation - point biserial model to measure the association between different levels of exposure and the changes of microplastics on nasal and gut microbiota. We chose the large ρ as 0.5, an α error prob as 0.05, and a power (1 – β error prob) as 0.90. The total sample size was calculated as 34. With a lost-to-follow-up rate of 10%, the number of sample size was set to 38 participants. We estimated that a sample of 40 participants is enough to explore the microbiota characteristics for the study.

### 2.4. Data and sample collection

All the data of the study will be collected and managed by Chinese Clinical Management Public Platform (http://www.medresman.org.cn/login.aspx) which records the management process of clinical trials, baseline data of subjects recorded during the trial, result data and other relevant data based on internet, and upload them to the central database for preservation and management. The data of this study can only be accessed and operated by the research team. Once the data are entered and stored, any changes made to the data will be automatically displayed and tracked. The public can view the related public information through “Public Browsing” when the enrollment is finished, but they will not be able to trace any personal information of the participants.

All members who collect samples and data will receive necessary training to make sure the accuracy of data. Anthropometric characteristics (including height without shoes, fasting weight in the morning) will be finished right after the enrollment. And we will recheck all the collected data at the end of the collecting. If there’s any missing data (such as height of some participants), we will contact the certain participant to fix it. If there’re some participants with missing data we cannot reach, we will record the number of participants with missing data for each variable of interest.

Researchers must wear disposable sterile gloves when collecting biological samples to avoid contacting with the actual sampling area and its surrounding area. If the glove is contaminated, it must be changed immediately. The study involved the collection and storage of biological specimens. All biological specimens will be destroyed right after use.

#### 2.4.1. Collecting specimens of feces.

Participants will receive necessary training on how to collect their own feces after enrollment. They will receive prepared cryopreservation box, marked sterile feces collector and sterile glass vials containing 5 mL of 75% medical alcohol in advance. Among them, samples in feces collector will be used for 16S rDNA sequencing, and samples in glass vials will be used for microplastic composition analysis.

Feces of each participant will be collected by sterile spoon (contained within the feces collector) and placed in the sterile feces’ collector and sterile glass vials respectively. These samples will be asked to store in the cryopreservation box immediately. And every participant should inform the research team right after the collection. Members of our group will be responsible for transmitting those samples. We will put the sterile feces collector in liquid nitrogen for 4 hours and then transfer it to −80°C for storage. And we will put sterile glass vials into 4°C to store.

#### 2.4.2. Collecting specimens of nasal secretions.

Researchers who are responsible for collecting nasal samples will receive necessary training on how to collect nasal secretion samples. The procedure we will perform is shown as below: first, gently rotate and wipe with a sterile cotton swab on the mucosal layer of the superior turbinate (about 2 cm) of the nasal cavity 2 to 3 times with the help of nasal endoscope; second, withdraw the swab slowly and place it into a sterile cryopreservation tube immediately; third, combine the left and right nasal samples of each participant into one; last, put them in liquid nitrogen for 4 hours and then transfer to −80°C for storage.

#### 2.4.3. Collecting environmental specimens.

**Figure 3.**
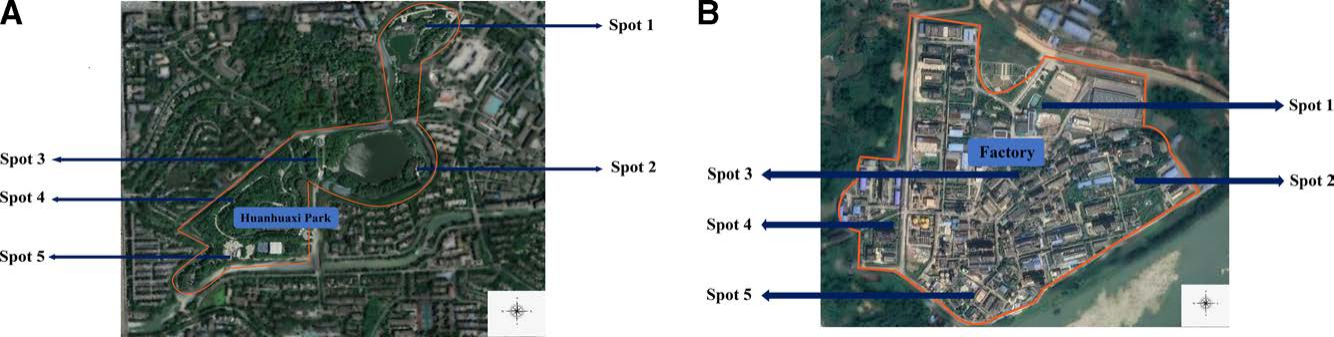
Map of sampled area. (A) Huanhuaxi Park, (B) plastic factory.

We will collect soil and air samples within 1 km from the center of the Huanhuaxi Park and at the plastic factory. Samples will be collected at 5 spots including the east, west, south, north, and center of the selected area (Fig. 3). The topsoil (10 cm) at 5 sampling points will be collected using a stainless steel sampling shovel.^[15–17]^ Those samples will be mixed uniformly to be one composite sample and wrapped by aluminum foil into a sampling bag.^[17]^ Before testing, we will place the samples in a clean, dry, dark place and store them at a low temperature. Outdoor air will be collected through an active suction sampler (MiniVolTM, Airmetrics, USA), which will be on for 6 to 8 hours at an adult’s breathing height (1.5 m) at 5 L/min.^[18,19]^

### 2.5. Outcome measures

We will collect samples and questionnaires right after the enrollment. Every participant will take assessment only one time.

#### 2.5.1. Primary outcome measures.

Diversity of nasal and gut microbiota analyzed by 16S rDNA sequencing (Illumina Hiseq 2500 sequencing platform, Biomarker Technologies Corporation, Beijing, China).

Microplastics from feces and environmental samples analyzed by 8700 LDIR laser infrared imaging (Agilent Technologies Co., Ltd, USA).

#### 2.5.2. Secondary outcome measure.

We will investigate the anthropometric characteristics of the study group, which can include height without shoes, fasting weight in the morning, and so on.

### 2.6. Data management and monitoring

Members of the research group will be responsible for collecting data. One statistician and 1 medical staff with physician certificate will be responsible for monitoring data. We will collect Case Report Forms which includes the basic information of participants. Two members of the research group (H.Y.C., X.Z.Y.) will enter collected information into the Chinese Clinical Trial Management Public Platform under confidential condition. The hard copy records will be preserved at a locked office. No one will be able to change or use the hard copy and electronic data without the authorization of our group.

## 3. Statistical methods

We will analyze quantitative data (age, working year, score of questionnaires, etc) to obtain each variable’s average (arithmetic mean, geometric mean, or median up to the distribution), standard deviation, interquartile range, etc. So that we can find out if background characteristics of the study group would be different from the volunteer group around the Huanhuaxi Park. We will also analyze qualitative data for variables such as gender distribution and proportion of each microbe species. And we will identify the core species based on the proportion above. We will compare the dichotomous variables between the 2 groups or among participants in the study group with or without certain microbe species using chi-square test or Fisher exact test if the theoretical frequency is < 1. We will compare the continuous variables between the 2 groups using *t* test or Wilcoxon signed-rank test if the distribution is not normal. We will also perform analysis to check the microbiota alteration among the 3 subgroups and compare it with the control group. We will perform multiple logistic regression for categorical variables and multiple linear regression for quantitative data to find out the potential predictors. We’re expecting to figure out how high exposure to microplastics would alter the structure and characteristics of microbiota. SPSS 24.0 software will be used to perform statistical analysis. *P* < .05 will be considered as statistically significant.

## 4. Discussion

Microplastics is one of those inhalable particles which can enter human body through respiratory tract. Digestive tract is considered as another major path for microplastics to enter human body,^[20]^ in which gut is the most gathering part. As the most intelligent creature in this planet, human beings can dominate the food chain, but also undergo much greater risks to intake more microplastic because of bioconcentration.^[21,22]^ It’s estimated that the amount of microplastics consumed by one American resident per year is 39,000 to 52,000.^[23]^ *United European Gastroenterology Week* in 2018 indicates that human feces contain microplastics with an average of 20 microplastics/10 g feces and a size of 50 to 500 μm. The current animal studies shows that exposure to microplastics can lead to the imbalance of gut microbiota of mice.^[11,13,24–27]^ Specifically, firmicutes increased significantly, while bacteroidetes and proteobacteria decreased significantly in those mice. We all know that it’s crucial to maintain the balance of microecosystem of nasal and gut for human health. More and more researchers are starting to realize the harmfulness of microplastics.

However, the current research on microplastics is still dominated by animal experiments, and the impact of microplastics on human health is still in its infancy, so relevant research is urgently needed. This study will conduct an observational cross-sectional study to investigate how microplastics affect the nasal and gut microbiota of high-exposure populations. We hope to build on these findings to provide solid evidence for filling the gaps in the effects of microplastics on human health. However, there’re still some limitations of this study. First, participants in study group will all come from 1 factory, which may not be able to represent the whole high-exposure population. Second, we collect environmental samples from multiple locations, yet the current collection standards are not completely unified, which may make our regional samples results have a certain degree of deviation.

## Acknowledgments

We would like to thank all participants for their support and contributions to this study. We also thank Cao Zhiqing, a statistician from Chengdu University of Traditional Chinese Medicine, for his guidance on this study.

## Author contributions

LY designed the study protocol and contributed to the subject recruitment. ZXY designed and wrote the study protocol and contributed to the data collection, laboratory measurement, and analysis. HYC contributed to write the study protocol and assist in the data collection and analysis. XZY performed laboratory measurements. PSH, WHT, and LL performed statistical analysis. XCG contributed to the study design, revised and edited the manuscript. KJ assisted in subject recruitment and data collection. YHP assisted in subject recruitment and data collection. All authors read and approved the final manuscript.

**Conceptualization:** Xiyu Zhang, Chunguang Xie, Ya LIU

**Data curation:** Xiyu Zhang, Yuchi He, Ziyan Xie, Haipo Yuan, Jian Kang, Ya LIU,

**Formal analysis:** Xiyu Zhang, Yuchi He, Sihan Peng, Heting Wang, Lu Liu

**Project administration:** Xiyu Zhang

**Writing – original draft:** Xiyu Zhang, Yuchi He

**Writing – review & editing:** Chunguang Xie

## Supplementary Material


