# Supplementary material for: Effect of microplastics on nasal and gut microbiota of high-exposure population: Protocol for an observational cross-sectional study
Source: Medicine (Baltimore). 2022 Aug 26;101(34):e30215. doi: 10.1097/MD.0000000000030215 (PMC9410575; doi:10.1097/MD.0000000000030215)
Supplement: Supplementary file 2 [file medi-101-e30215-s002.pdf]

Informed Consent Form

Name of participant: Gender: Age: Registry No:

Dear participant,

The purpose of this study is to fill the gap in the impact of microplastics on human health and to explore the characteristics of the nasal cavity and intestinal microbiota of people with high exposure to microplastics.

This study is designed as a cross-sectional study. If you agree to participate, you will need to: (a) fill out some questionnaires and answer some questions to assess your health status, (b) receive a collection of nasal secretions, (c) collect fresh intestinal secretions. Before collection, you will receive training on how to collect samples.

The participation in this study is entirely voluntary. Participants may be withdrawn at any time during the study without affecting the relationship between you and researchers. There is no loss in economic aspect for you.

The research will strictly protect your privacy according to the principles of the Declaration of Helsinki. All information in this study will be kept confidential, and your private information will not appear in the research summary and published literature. This study has been ethically reviewed by the Chinese Ethics Committee of Registering Clinical Trials (Ethical review document number: ChiCTR2100049480).

Voluntary Subject Statement

I have learned about the requirements of the observational study in detail and the potential risks or discomforts associated with it. I voluntarily participate in this observational study and fill out the relevant health questionnaire in accordance with the requirements of the research program. If there is any discomfort, I will report to the researcher in time. At the same time, I know that the researcher will give positive response for my possible discomfort. I am also entitled to withdraw from the study at any time for any reason. However, if there are no special circumstances, I will cooperate with the researcher to complete the observational study. My participation and the personal data in the trial are confidential. I agree with my researcher, the relevant regulatory authorities, and the ethics committee to review my information as required.

I (signature) relative (signature) (Relationship)

Date: D M Y

Researcher Declaration

I have fully elaborated the requirements of the observational study in detail and the potential risks or discomforts to the above participant/relative and answered their questions. To the best of my knowledge, the participant/relative has been informed adequately and has consented.

Researcher’s signature Date: D M Y

In the event of inconsistency or discrepancy between the Chinese version and the English version, the Chinese language version shall prevail.
